# Obesity, incretin-based therapies, and cardiovascular disease in women

**DOI:** 10.1007/s12471-026-02084-0

**Published:** 2026-09-16

**Authors:** Miro Salih, Yannicke A. J. Sloots, Martin E. W. Hemels, Laura M. G. Meems, Maarten J. G. Leening, Chahinda Ghossein-Doha

**Affiliations:** 1 Department of Cardiology, Cardiovascular Institute, Erasmus MC—University Medical Center Rotterdam, Rotterdam, The Netherlands; 2 Department of Cardiology, Rijnstate Hospital, Arnhem, The Netherlands; 3 Department of Cardiology, Radboud University Medical Centre, Nijmegen, The Netherlands; 4 Department of Cardiology, University Medical Center Groningen, University of Groningen, Groningen, The Netherlands; 5 Department of Radiology and Nuclear Medicine, Erasmus MC—University Medical Center Rotterdam, Rotterdam, The Netherlands; 6 Department of Epidemiology, Erasmus MC—University Medical Center Rotterdam, Rotterdam, The Netherlands; 7 Department of Cardiology, Hartcentrum Hasselt and Department of Jessa and Science, Jessa Hospital, Limburg Clinical Research Center, Hasselt, Belgium; 8 Faculty of Medicine and Life Sciences, UHasselt, Limburg Clinical Research Center, Diepenbeek, Belgium

**Keywords:** Obesity, Women, Glucagon-Like Peptide-1 Receptor Agonists, Cardiovascular disease, Adiposity, Heart Failure, Menopause, Pregnancy

## Abstract

Obesity in women is a growing and multifaceted public health challenge, driven by complex interactions among biological, hormonal, and psychosocial factors across the life course. From menarche to menopause, hormonal fluctuations influence fat distribution, energy balance, and metabolic risk, contributing to sex-specific trajectories of weight gain. Moreover, women-specific conditions, such as pregnancy-related weight retention, hypertensive disorders of pregnancy, and polyendocrine metabolic ovarian syndrome, further amplify long-term cardiometabolic risk. Women with obesity have an increased risk of heart failure with preserved ejection fraction and ischemia with non-obstructive coronary arteries.

Emerging pharmacological treatments for obesity, including incretin-based therapies, offer an evidence-based addition to lifestyle interventions and metabolic and bariatric surgery and have demonstrated substantial weight loss effects and cardiovascular benefits. However, sex-specific differences in efficacy, tolerability, and long-term outcomes of these new pharmacological treatments remain insufficiently researched. Moreover, concerns regarding reproductive health and access to care uniquely affect women. This review addresses the need for a life course and sex-specific approach to obesity and its treatment, integrating metabolic, reproductive, and cardiovascular perspectives.

## Introduction

Obesity, defined as a body mass index (BMI) > 30 kg/m^2^, has emerged as a leading global health challenge and a major driver of cardiovascular disease (CVD), with a disproportionate impact on women. According to the World Health Organization, more than 890 million adults were living with obesity in 2022, corresponding to approximately 16% of the global adult population [1]. Higher obesity rates are observed in women compared to men (18% vs 14%), and prevalence rates are rising in almost every country [1]. Obesity is a key risk factor for atherosclerotic CVD and heart failure (HF), yet its cardiovascular consequences differ between the sexes due to biological and sociocultural factors [2–5]. Accordingly, the cardiometabolic risk profile differs between women and men, partly due to sex-specific fat distribution and hormonal interactions [6–8]. Women preferentially accumulate subcutaneous adipose tissue, whereas men exhibit increased visceral tissue [7, 8]. Both subcutaneous and visceral fat tissue are associated with health risks. However, subcutaneous adipose tissue is less strongly associated with cardiometabolic risk factors, such as hypertension and impaired fasting glucose, compared to visceral fat tissue [7].

Hormonal transitions across a women’s life course critically shape obesity trajectories. Pregnancy-related complications and the menopausal transition contribute to adverse shifts in fat distribution and metabolic risk. These shifts are, in turn, associated with conditions such as microvascular dysfunction and heart failure with preserved ejection fraction (HFpEF) [9–11].

Lifestyle intervention is the cornerstone of obesity treatment and can be complemented with metabolic and bariatric surgery or a pharmacologic agent for sustained weight loss and reduction in obesity-related adverse outcomes [12]. In the Netherlands, guidelines recommend metabolic and bariatric surgery for individuals with a BMI above 40 kg/m^2^, with BMI of 35–39.9 kg/m^2^ and a related comorbidity, or a BMI above 30 kg/m^2^ accompanied by dysregulated type 2 diabetes mellitus (T2DM). Obesity management is reshaped by the rise of incretin-based therapies, such as Glucagon-like peptide‑1 receptor agonists (GLP-1RAs) and dual GLP-1RA and Glucose-dependent insulinotropic polypeptide (GIP) receptor agonists (GLP-1RA/GIP), which consistently demonstrate improved glycated haemoglobin levels and substantial weight loss. In the Netherlands, GLP-1 RA liraglutide is reimbursed for high CVD risk T2DM, and combined with lifestyle intervention for BMI above 40, or BMI above 35 with a comorbidity. Tirzepatide, a GLP-1RA/GIP, has been shown to reduce weight by 21%, compared to 3% with placebo [13]. Additionally, studies with incretin-based therapies have demonstrated significant reduction in adverse cardiovascular events and death among patients with obesity or overweight and established CVD [14, 15]. However, sex-specific differences in efficacy, tolerability, and cardiovascular benefit remain insufficiently researched [16].

In this review, we examine obesity in women, the associated cardiovascular risks and treatment strategies, with a specific focus on incretin-based therapies, and provide insights into how a women-specific, life-course approach can enhance cardiovascular prevention and improve long-term health outcomes.

Tab. 1 summarizes key domains relevant to obesity and cardiovascular risk in women, spanning epidemiology, adipose tissue biology, hormonal regulation, life-course transitions, metabolic disease, cardiovascular phenotype, pharmacological therapy, reproductive considerations, sociocultural factors, and healthcare system factors, alongside current evidence and implementation gaps, to guide future research and clinical practice.

**Table 1:** Key sex-specific considerations and clinical gaps at the intersection of obesity, metabolic disease, and cardiovascular health in women

Domain	Key factors in women	Current gaps
Epidemiology & risk	Higher obesity prevalence in women (≈ 18% vs 14%); earlier onset in socioeconomically vulnerable groups	Under-recognition of obesity as primary CV risk factor in women
Adipose tissue biology	Predominantly subcutaneous fat; transition to visceral fat with menopause	Limited integration into risk prediction models
Hormonal regulation	Oestrogen effects; PMOS (androgen excess); menopause-related fat redistribution	Lack of hormone-informed obesity management strategies
Life course transitions	Pregnancy, postpartum weight retention, (premature) menopause	Limited incorporation into CV prevention guidelines
Metabolic disease	Strong link with insulin resistance and T2DM; higher relative CV risk in women with T2DM (~40% higher vs men)	Underestimation of diabetes-related CV risk in women
Cardiovascular phenotype	Coronary microvascular dysfunction; higher prevalence INOCA; HFpEF	Gap between sex-specific research and routine implementations in the clinics
Pharmacological therapy	Greater weight loss response to GLP-1RA; higher adverse effects	Underrepresentation of women in trials; lack of sex-stratified analyses
Reproductive considerations	Pregnancy safety, fertility, contraception, breastfeeding	Major evidence gap in drug safety in reproductive-age women
Sociocultural factors	Weight stigma, healthcare bias, caregiving roles	Rarely addressed in clinical guidelines
Healthcare system factors	Fragmented care across cardiology, endocrinology, and gynaecology	Lack of integrated care models

*CV* cardiovascular, *PMOS* polyendocrine metabolic ovarian syndrome, *T2DM* type 2 diabetes mellitus, *INOCA* ischemia with non-obstructive coronary arteries, *HFpEF* heart failure with preserved ejection fraction, *GLP-1RA* glucagon-like peptide‑1 receptor agonist

## Epidemiology and cardiometabolic implications of obesity

### Sex differences in epidemiology

Prevalence of obesity is steadily increasing across the globe over recent decades [1] and is slightly higher among women than among men (Global: 18% vs 14%, Europe: 24% vs 22%) [17]. Obesity rates are higher among people with lower educational attainment, lower income, and less differentiated occupations [18]. The link between socioeconomic status and BMI is more consistent for women than for men; the association between living in socioeconomically disadvantaged neighbourhoods and obesity is stronger in women than in men [2]. These data point towards sex-related discrepancies shaped not only by biological factors, but also by determinants such as socioeconomic status.

### Obesity in women and cardiovascular risk

**Fig. 1:**
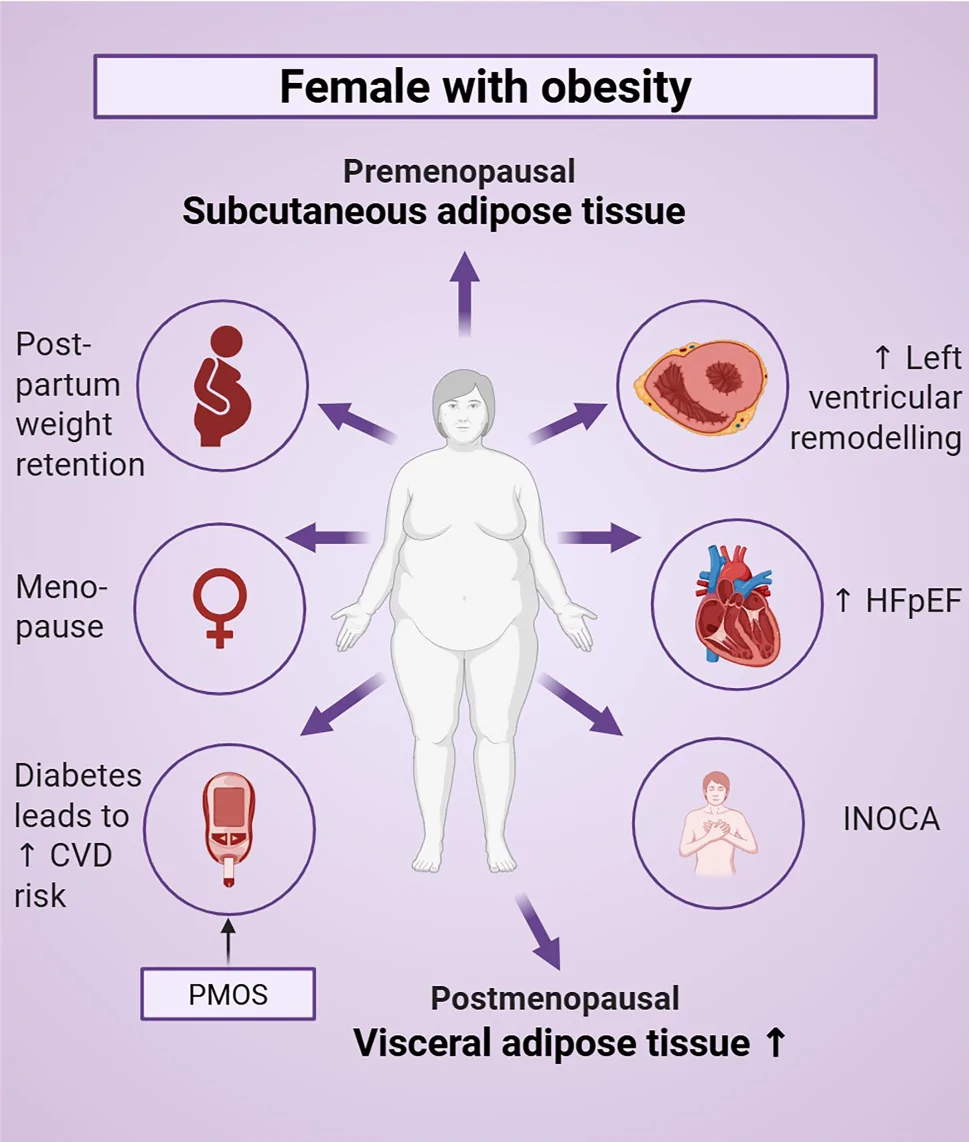
Clinical considerations of obesity in women. Premenopausal women accumulate more subcutaneous adipose tissue, while postmenopausal women show increased visceral adipose tissue. In women, obesity is associated with an increased risk for left ventricular remodelling, HFpEF and INOCA. Pregnancy affects body composition. Menopause leads to elevated lipid levels and increased prevalence of metabolic syndrome. Diabetes mellitus leads to increased CVD risk in women compared to men. PMOS affects diabetes risks. *HFpEF* heart failure with preserved ejection fraction, *INOCA* ischemia with non-obstructive coronary arteries, *CVD* cardiovascular disease, *PMOS* polyendocrine metabolic ovarian syndrome

Obesity is strongly associated with an increased CVD risk. Large-scale cohort studies have demonstrated that each 5 kg/m^2^ increase in BMI starting with a BMI above 25 kg/m^2^, is associated with an approximately 40% higher hazard ratio of coronary heart disease (CHD) and stroke [3]. The CVD risks associated with obesity in women (Fig. 1) appear to differ from those in men, both in terms of the type of risk and the extent of its impact. In an analysis from the Framingham Heart Study, it was reported that the excess risk of CVD attributed to obesity was 46% in men, compared to 64% in women [5]. Specifically, with greater BMI and waist circumference, women show a steeper decline in echocardiographic measures of adverse left ventricular remodelling, compared to men [5]. Atrial fibrillation is more prevalent in men than in women, and this sex difference is more pronounced at higher BMI [19]. Among women, obesity is more strongly associated with the development of HFpEF than HF with reduced ejection fraction (HFrEF), whereas the opposite pattern is observed in men with obesity [4].

## Sex-specific biological mechanisms linking obesity to CVD

### Adipose tissue distribution and sex hormones

Sex-specific differences in adipose tissue distribution and function play a central role in modulating cardiometabolic risk. Men exhibit greater visceral adiposity (also known as upper body adipose tissue distribution), whereas young, premenopausal women generally accumulate more subcutaneous adipose tissue, particularly in the gluteofemoral region (lower body distribution). Visceral adipose tissue is more strongly associated with insulin resistance, dyslipidaemia, and systemic inflammation [7, 20]. This likely results from the unfavourable metabolic activity of visceral adipose tissue, including the secretion of adipocytokines and other vasoactive substances [7].

Sex hormones are key regulators of adipose tissue distribution, energy homeostasis, and cardiometabolic risk in women, contributing to distinct obesity trajectories and cardiovascular risk across the life course. From puberty onwards, oestrogen promotes preferential subcutaneous fat deposition, which is more insulin-sensitive, less susceptible to inflammation, and has increased mitochondrial activity [8]. Consequently, these sex differences in adipose tissue distribution and function act as drivers for the differences observed in obesity-related metabolic risk between men and women.

### Pregnancy

Pregnancy represents a critical metabolic stress test, during which physiological insulin resistance and fat accumulation are necessary to support foetal development. However, excessive gestational weight gain and postpartum weight retention are common, with approximately 75% of women retaining significant weight one year after delivery (Fig. 1; [21]). In addition, pregnancy complications such as gestational diabetes and hypertensive disorders of pregnancy, including preeclampsia, are strongly associated with obesity, T2DM, and an increased risk of CVD later in life [5, 22]. These conditions may unmask an underlying predisposition to cardiometabolic disease, positioning pregnancy as an early window of opportunity for risk stratification and intervention.

### Menopause

The menopausal transition occurs around age 50 and is accompanied by significant changes in body composition (Fig. 1; [10]). Declining oestrogen levels are associated with increased visceral adipose tissue accumulation and reduced energy expenditure, resulting in an annual weight gain of approximately 0.3–0.4% during the transition [23, 24]. Central adiposity increases significantly across the menopausal transition; an increase of 4.63 cm in waist circumference (a visceral/central fat measure) has been reported in postmenopausal compared with age-matched premenopausal women [9, 25]. Lipid profiles also change during menopause; LDL-cholesterol and triglycerides levels increase by 10–15%, whereas HDL cholesterol levels decrease slightly [10]. These shifts are thought to result largely from declining oestradiol and oestrogen levels, which play important roles in metabolic and enzymatic activity. Reduced oestradiol levels increase the activity of lipolytic enzymes, such as hepatic lipase, affecting the HDL metabolism [26]. Oestrogens have been shown to suppress the transcription of lipoprotein lipase, which may contribute to LDL-cholesterol changes associated with menopause [27], but further research into these mechanisms is needed. Together, these changes are reflected in a 2- to 3‑fold higher prevalence of metabolic syndrome, a cluster of conditions including excess abdominal adiposity and elevated fasting glucose, in postmenopausal women compared to premenopausal women of the same age [5, 10]. As a result, menopause is associated with a marked increase of cardiovascular risk. This is underscored by evidence that premature menopause (before 45 years of age), affecting up to 10% of women, is associated with a 1.5-fold higher risk of CHD [28].

### Obesity and menopausal symptoms

**Fig. 2:**
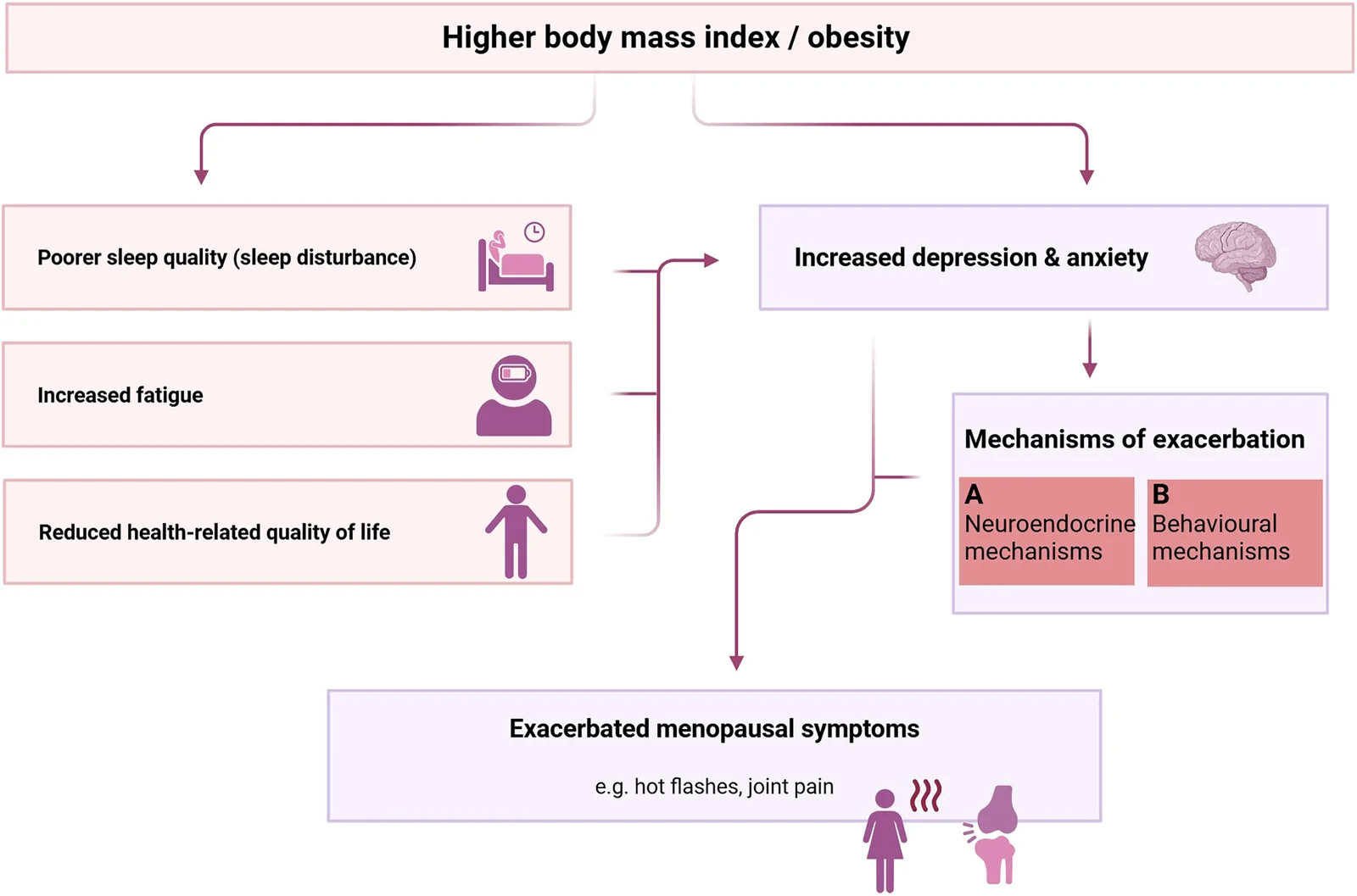
Pathways linking obesity to the exacerbation of menopausal symptoms. Increased BMI leads to poorer sleep quality, increased fatigue, and reduced health-related quality of life. Moreover, obesity is strongly linked to depression and anxiety, which might further exacerbate menopausal symptoms (hot flashes, joint pain) through neuroendocrine and behavioural mechanisms

In perimenopausal women, increased adiposity is associated with greater frequency and severity of vasomotor symptoms. Observational studies indicate that women with overweight and obesity have a high likelihood of experiencing hot flashes and night sweats during the menopausal transition [29], thought to be mediated by impaired thermoregulation and altered hypothalamic signalling, compounded by inflammatory pathways linked to excess adipose tissue. Higher BMI is also associated with poorer sleep quality, greater fatigue, and reduced health-related quality of life during the menopausal transition (Fig. 2; [30]). Moreover, obesity is strongly associated with depression and anxiety, which may further exacerbate menopausal symptoms through neuroendocrine and behavioural mechanisms (Fig. 2; [31]).

Menopausal symptoms, particularly sleep disturbance, fatigue, and mood disorders, may promote weight gain through reduced physical activity and adverse dietary behaviours, creating a bidirectional relationship between menopausal symptoms and obesity. Together, these findings highlight a self-reinforcing cycle in which hormonal changes, adiposity, metabolic dysfunction, and symptom burden interact to accelerate cardiovascular risk in midlife women. Strategies that simultaneously address menopausal symptoms, weight management, and CVD prevention could help address an important unmet clinical need.

### Obesity and metabolic dysfunction

Obesity is a major driver of metabolic disease, particularly T2DM and metabolic dysfunction-associated steatotic liver disease (MASLD). Up to 90% of individuals with T2DM have overweight or obesity [32]. The relationship between obesity and T2DM is bidirectional and influenced by sex-specific factors, including hormonal regulation. In women specifically, there is an interplay between obesity and conditions characterized by hormonal imbalance, such as polyendocrine metabolic ovarian syndrome (PMOS), in which androgen levels are elevated. PMOS further amplifies the risk of insulin resistance and T2DM [33]. Additionally, women with T2DM have a higher relative risk of CVD than men with T2DM (~40% higher risk of CHD) [5].

MASLD, a group of diseases characterized by fat accumulation in the liver, occurs in 75% of people with obesity [34]. This prevalence of MASLD is higher in men than in premenopausal women, but after menopause the prevalence is higher in women, suggesting an important role for hormonal factors [34]. Both overnutrition and obesity increase MASLD risk through mitochondrial dysfunction and activation of inflammatory pathways [35]. In turn, MASLD leads to a substantially increased risk of atherosclerosis, HF, and also T2DM.

### Cardiac and microvascular remodelling

Obesity is associated with cardiac and microvascular remodelling, an association that seems stronger in women than in men. The prevalence of diastolic dysfunction is higher in women than in men with metabolic syndrome, a condition closely linked to central obesity [36]. Obesity is increasingly recognized as a key contributor to coronary microvascular dysfunction (CMD), a disorder affecting the structure and function of the coronary microcirculation, with a positive correlation between increasing BMI and CMD severity [37].

The mechanisms linking obesity and CMD have not yet been fully elucidated, although research shows that the pro-inflammatory and insulin-resistant state promoted by visceral adipose tissue leads to increased vascular oxidative stress and reduced nitric oxide bioavailability [38, 39]. Cytokines and adipokines released by adipose tissue, such as leptin and adiponectin, further modulate vascular function and stimulate myocardial fibrosis [37]. Women appear particularly vulnerable to these mechanisms because of sex-specific differences in vascular biology, including smaller coronary vessel size, heightened microvascular reactivity, and hormonal fluctuations [40]. The decline in oestrogen during menopause exacerbates endothelial dysfunction and promotes microvascular impairment.

## Obesity-related cardiovascular conditions in women

Cardiovascular conditions associated with obesity in women include ischaemia with non-obstructive coronary arteries (INOCA, a condition characterized by anginal chest pain in the absence of obstructive coronary artery disease) and HFpEF. INOCA is more common in women than in men, with high prevalence especially among ages 45–65 years [5]. The mechanisms underlying anginal symptoms in INOCA are not fully understood. One of the proposed mechanisms is CMD, which predominantly affects women (60–70% of patients) [5]. Traditional risk factors, such as hypertension, insulin resistance, hyperlipidaemia and obesity, are associated with INOCA in the general population and remain important risk factors in women [37]. Importantly, CMD and INOCA are associated with adverse cardiovascular outcomes, including HF, particularly HFpEF, and recurrent hospitalizations [41]. CMD is present in 75% of patients with HFpEF [9].

Accordingly, obesity has been associated with the development of HFpEF in women, likely through increased inflammation and signalling pathway dysregulation, leading to endothelial dysfunction, mitochondrial impairment, and myocardial injury [9, 36].

**Fig. 3:**
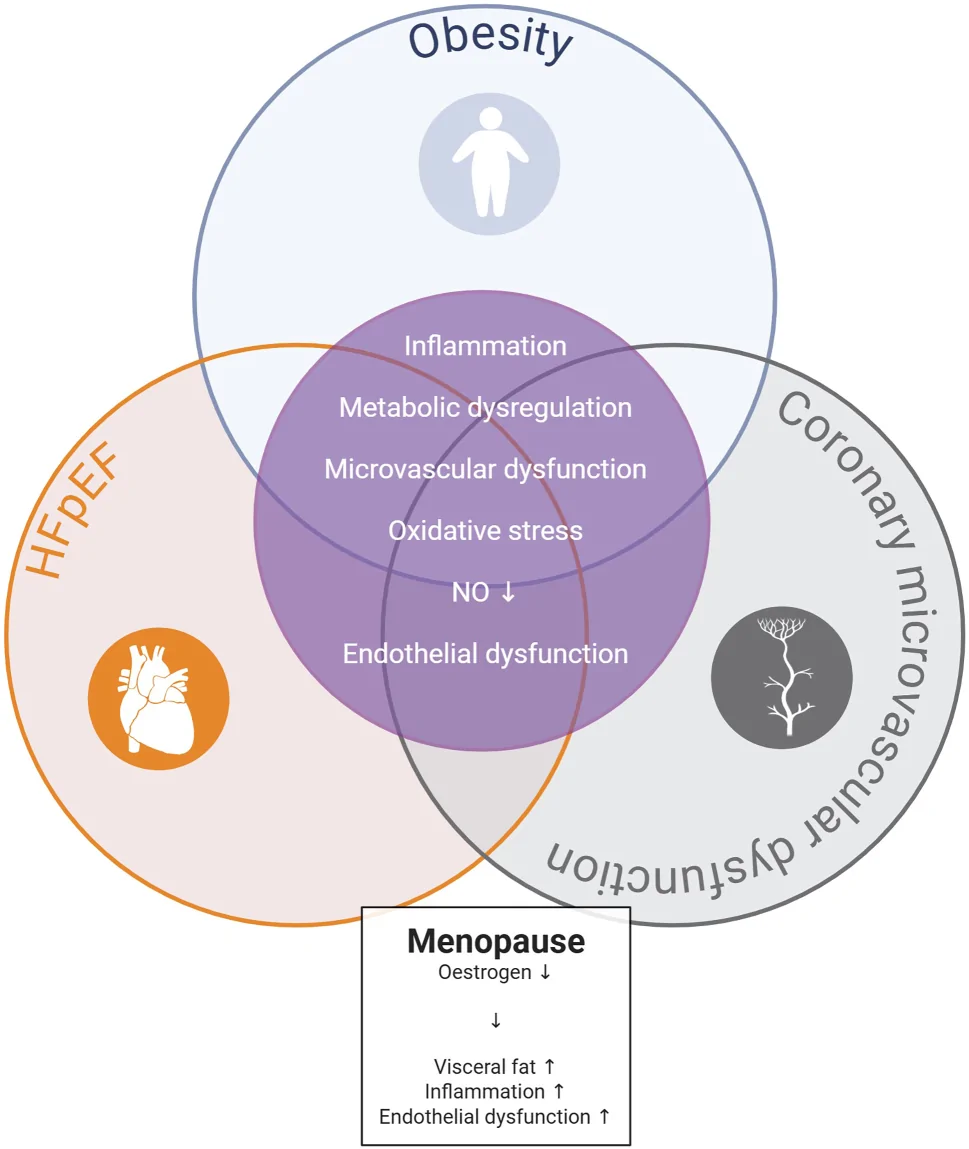
Pathophysiological overlap between obesity, microvascular dysfunction, and HFpEF in women. A systemic syndrome driven by inflammation, metabolic dysregulation, microvascular dysfunction, oxidative stress, decreased NO, and endothelial dysfunction is shared between the three conditions. The increase in visceral adipose tissue during menopause further contributes to these pathways. *HFpEF* heart failure with preserved ejection fraction, *NO* nitric oxide

Obesity, CMD, and HFpEF are closely linked and may represent different clinical expressions of a common underlying systemic process involving inflammation, metabolic dysfunction, and microvascular impairment (Fig. 3). This process may be further influenced by menopause, as declining oestrogen levels and relatively increased androgen concentrations are thought to promote vascular dysfunction, insulin resistance, and atherogenesis. Consequently, menopause is associated with a marked increase in the prevalence of atherosclerotic CVD, microvascular angina and HFpEF [9, 42].

## Therapeutic strategies and sex-specific considerations

Weight loss is the most important cornerstone in obesity and CVD risk management. Each one-kilogram loss in body weight has been associated with a 1 mm Hg reduction in systolic blood pressure [43] and a 16% reduction in T2DM risk [44], highlighting the effectiveness of weight loss. Lifestyle intervention, including dietary, physical activity, and behavioural changes, is the cornerstone of obesity treatment and can be complemented by pharmacotherapy and metabolic and bariatric surgery. Pharmacological strategies include orlistat, naltrexone-bupropion and incretin-based therapies (DPP-4 inhibitors, GLP-1RAs or dual GLP-1RA/GIP therapies) [45].

The use of incretin-based therapies has increased substantially. In recent years, dispensing of dulaglutide, exenatide, liraglutide, semaglutide or tirzepatide in adolescents and young adults in the US increased 594.4%, with 76% of young adults being female [46]. The mean percent weight change after 72 weeks with this drug class ranges from −14% with early-generation agents such as semaglutide, to −20% with more recent dual GLP1-RA/GIP therapy (tirzepatide) [47]. Beyond weight loss, this drug class has shown reduction in cardiovascular endpoints: the SELECT trial showed that semaglutide reduced the relative risk of death or cardiovascular event by 20% in individuals with overweight or obesity and established CVD, without T2DM [14]. The SUMMIT trial in patients with HFpEF (104-week follow-up) showed that tirzepatide lowers the risk of cardiovascular death or a worsening HF event compared to placebo (HR 0.62, 95%CI 0.41–0.95), with comparable benefit in women (HR 0.66, 95%CI 0.38–1.18) and men (HR 0.61, 0.33–1.13), and improved KCCQ-CSS, exercise tolerance (6-min walk distance) and C‑reactive protein (CRP) levels [15]. Dual agonist maridebart cafraglutide and triple-hormone-receptor agonist retatrutide further expand therapeutic possibilities [48, 49]; retatrutide achieved average weight loss up to around 24% after 48 weeks, compared to placebo [48]. In the general obesity population, these agents demonstrate favourable effects on blood pressure, glycated haemoglobin, and lipid profiles, suggesting broader cardiometabolic benefits; whether these translate into reduced cardiovascular events in patients without established CVD/HF remains to be tested in ongoing trials.

### Sex differences in incretin-based therapies

Several clinical trials have shown that incretin-based therapies result in significantly more weight loss in women than in men; in the SURMOUNT trial, women showed 6 percentage points higher weight loss compared to men [47] and similar results are also reported elsewhere [48]. There may be an altered pharmacodynamic response to GLP-1RA-based therapies in women, potentially reflecting an interaction between GLP-1RAs and oestrogen. Preclinical studies have shown that co-administration of a GLP1-RA with oestrogen activates the supramammillary nucleus, a brain region not activated by either compound alone [50], suggesting oestrogen may broaden or modify the central nervous system effects of these drugs [16]. Combined GLP1 and oestrogen receptor activation also enhanced metabolic benefits (improved energy, glucose and lipid metabolism), which could contribute to greater weight loss [51]. Beyond this, the downstream pathways engaged by GLP-1RAs appear to differ between men and women: preclinical studies show that GLP1receptor activation suppresses orexin (an orexigenic neuropeptide) signalling only in females, whereas the interleukin response was more potent in males [52]. Lastly, clinical data showed that exenatide (combined with metformin) produced greater improvement in inflammatory and adipokine markers (CRP, TNF‑α, adiponectin) in women than men, suggested to further contribute to weight loss [53].

Importantly, sex-specific considerations remain insufficiently addressed for incretin-based therapies. Sex-stratified analyses are often limited. Available data suggest women report higher rates of gastrointestinal adverse effects and headache, which may negatively affect adherence and persistence [16, 54]. The impact of these therapies on reproductive health, pregnancy and neonatal outcomes also remains inadequately studied, limiting their integration into routine care for women of reproductive age.

Beyond biological differences, gender-related factors, including weight stigma and healthcare bias, continue to influence treatment uptake and effectiveness. Women are more likely to experience weight-related stigma, associated with delayed healthcare-seeking behaviour, reduced treatment adherence, and adverse mental health outcomes [55].

## Conclusion and future perspectives

This review provides an overview of obesity and CVD in women, with key concepts and priority areas for future research and clinical practice outlined in Tab. 1. Sex differences in the relationship between adiposity and CVD between men and women, such as increased risk of HFpEF in women with obesity, are increasingly acknowledged and studied. Women-specific life events, such as pregnancy and menopause, represent windows of increased cardiovascular risk and should prompt closer monitoring and, where indicated, earlier intervention than in men, who do not experience comparable hormonally driven risk transitions.

The advent of progressively effective obesity therapies marks a potential turning point in cardiovascular medicine. Recent trials provide compelling evidence that GLP-1RAs reduce cardiovascular risk, supporting a paradigm shift towards obesity as a primary therapeutic target. However, critical gaps persist in understanding sex-specific responses and optimal integration across different stages of a woman’s life. In particular, the lack of data on reproductive health, pregnancy safety, and potential menopause-related effects on therapy represents a significant knowledge gap. Future research should prioritize sex-specific analyses in clinical trials, including adequate representation of women across age groups and reproductive stages. Standardized data collection in registry studies of women using specific drug classes during the periconceptional period and pregnancy is essential to assess safety in reproductive age. Longitudinal, life-course studies are needed to better understand critical windows for intervention, particularly around pregnancy and menopause, essentially paving the way to improve therapeutic utilization in both men and women.

## Data Availability

All data supporting the findings of this work are included in the article.
